# Equal Z standard-setting method to estimate the minimum number of panelists for a medical school’s objective structured clinical examination in Taiwan: a simulation study

**DOI:** 10.3352/jeehp.2022.19.27

**Published:** 2022-10-17

**Authors:** Ying-Ying Yang, Pin-Hsiang Huang, Ling-Yu Yang, Chia-Chang Huang, Chih-Wei Liu, Shiau-Shian Huang, Chen-Huan Chen, Fa-Yauh Lee, Shou-Yen Kao, Boaz Shulruf

**Affiliations:** 1 Division of Clinical Skills Training Center, Taipei Veterans General Hospital, Taipei, Taiwan; 2 National Yang-Ming University, Taipei, Taiwan; 3 University of New South Wales, Sydney, Australia; 4 Department of Medical Education, Taipei Veterans General Hospital, Taipei, Taiwan; 5 University of Auckland, Auckland, New Zealand; Hallym University, Korea

**Keywords:** Computer simulation, Educational measurement, Standards, Taiwan, Undergraduate medical education

## Abstract

**Purpose:**

Undertaking a standard-setting exercise is a common method for setting pass/fail cut scores for high-stakes examinations. The recently introduced equal Z standard-setting method (EZ method) has been found to be a valid and effective alternative for the commonly used Angoff and Hofstee methods and their variants. The current study aims to estimate the minimum number of panelists required for obtaining acceptable and reliable cut scores using the EZ method.

**Methods:**

The primary data were extracted from 31 panelists who used the EZ method for setting cut scores for a 12-station of medical school’s final objective structured clinical examination (OSCE) in Taiwan. For this study, a new data set composed of 1,000 random samples of different panel sizes, ranging from 5 to 25 panelists, was established and analyzed. Analysis of variance was performed to measure the differences in the cut scores set by the sampled groups, across all sizes within each station.

**Results:**

On average, a panel of 10 experts or more yielded cut scores with confidence more than or equal to 90% and 15 experts yielded cut scores with confidence more than or equal to 95%. No significant differences in cut scores associated with panel size were identified for panels of 5 or more experts.

**Conclusion:**

The EZ method was found to be valid and feasible. Less than an hour was required for 12 panelists to assess 12 OSCE stations. Calculating the cut scores required only basic statistical skills.

## Introduction

### Background/rationale

One of the most challenging tasks in assessment in high-stakes examinations in higher education is accurately differentiating between competent and incompetent examinees. To address this challenge, a common practice is to employ a standard-setting process that determines a cut score for the entire examination, or parts of the examination if the assessment is composed of multiple independent sections [1]. Among the plethora of standard-setting methods, the most commonly used are methods that employ panels of experts who systematically assess the examination and items. Such techniques include, but are not limited to, the most popular Angoff method and its variants, the Ebel method, the bookmark method, the item mapping method (a variant of the bookmark method), and the Hofstee method [1,2]. Despite their popularity, the Angoff and the modified Angoff methods have attracted some critique. It has been suggested that experts are vulnerable to judgment biases [3,4]. It has also been suggested that the Angoff method requires a minimum of 15 experts per panel to yield reliable cut scores [2]. Moreover, the Angoff method is resource-heavy since it requires the panel to review and estimate the probability of each item being correctly answered by the minimally competent examinee, which commonly takes a few hours to complete [1]. Some new and improved methods have been introduced over the past decades [5-8], each with its own strengths and weaknesses.

The most recently introduced method, the equal Z method (henceforth: EZ method, pronounced “easy method”) aimed to generate cut scores that are placed between the average minimum passing score and the averaged maximum failing score for the entire examination as determined by a panel of experts [8]. The new feature presented in the EZ method is that its cut score is placed at the point set at the same distance from the minimum passing score and the maximum failing score, as measured by the respective z-scores around these 2 points. Although identical in terms of z-scores, they may be different in absolute values due to the different distribution of the scores around these 2 points. Evidence supporting the validity of the EZ method has already been presented [8], yet no previous study has aimed to estimate the minimum number of experts required to sit on the panel to yield reliable cut scores.

### The equal Z (EZ) method

The EZ method uses a panel of experts who work independently to assess the entire examination. In the case presented in this study, the examination consisted of 12 stations of an objective structured clinical examination (OSCE), a common high-stakes examination used in a range of health professions education and examination modes [5,9,10]. In the EZ method, each expert separately provides answers to the following 2 questions: first, what would be the lowest score that indicates, without any doubt, that an examinee is competent in the topics assessed?; second, What would be the highest score that indicates, without any doubt, that an examinee is incompetent in the topics assessed?

These scores are then used to calculate the cut scores for each of the stations using the following procedure:

For each station, we define L as the highest failing score below which an examinee is incompetent; and we define H as the lowest passing score above which an examinee is competent. From the collated scores (L and H), the means of L and H (XL and XH, respectively) and standard errors of the means (SEL and SEH, respectively) are calculated.

Equation 1 is used to identify the same Z score (Z) that would apply to both confidence intervals of X_L_ and X_H_ when they interface:

 

Equation 1

Z*SE_L_+Z*SE_H_=X_H_−X_L_

 

From Equation 1, we extract Z using Equation 2:

Equation 2

Z=(X_H_−X_L_)/(SE_L_+SE_H_)

 

The cut score is then set at X_L_+Z*SE_L_, which is also equal to X_H_ Z*SE_H_.

**Fig. 1.**
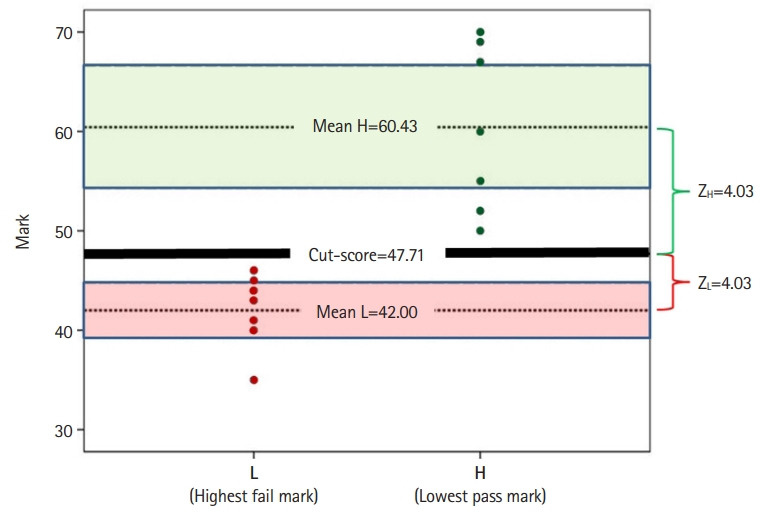
Demonstration of the equal Z method.

To illustrate how the EZ model works, data from a fictitious expert panel of 7 members are presented here: Each panelist provides the lowest pass mark “without any doubt” (H, green dots on the right, Fig. 1) and the highest failure mark (L, red dots on the left, Fig. 1). For each of the 7 H and L marks, the mean H (X_H_) and mean L (X_L_) were calculated (that is, 60.43 and 42.00, respectively). The standard errors for H (SE_H_) and L (SE_L_) were also calculated (8.34 and 3.74, respectively). Using the information of the 2 standard errors and means, equation 1 (Z*SE_L_+Z*SE_H_=X_H_−X_L_) is used to find Z, the point equidistant from the means. Extracting Z from equation 2 [Z=(X_H_−X_L_)/(SE_L_+SE_H_)=(60.43−42.00)/(3.74+8.34)] yields Z equal to 1.53. The cut score is then calculated using either X_H_−1.53*SE_H_ or X_L_+1.53*SE_L_. Both result in a cut score of 47.71. This suggests, with a confidence of 93.70% (since Z=1.53), that the cut score (47.71) is neither a pass (i.e. <60.43) nor a fail (i.e. >42.00) (Fig. 1).

Although the EZ method somewhat resembles the Hofstee method by being simple and light on resources, there are 2 main differences between the Hofstee and the EZ methods. First, the Hofstee method is a “compromise” method combining both norm- and criterion-referenced approaches, whereas the EZ method uses a criterion-referenced approach only. Second, the criterion-referenced questions asked in the 2 methods are significantly different. That is, the Hofstee method requires experts to estimate the highest and lowest acceptable percentage for correct and incorrect cut scores. Note that “acceptability” requires the experts to consider others’ perceptions. The EZ method, on the other hand, requires the experts to indicate “without any doubt” the highest failure marks and the lowest pass marks for the examination. The EZ method does not ask the experts to estimate any perceptions other than their own.

Previous studies have demonstrated that the EZ method requires experts to spend about 1 hour assessing 12 OSCE stations (equivalent to assessing an examination with 12 sections on different topics; each includes about 10–15 items, which equates to an examination comprising 120–180 items); and it yielded cut scores with high statistical confidence [7,8]. Nonetheless, the unanswered question relates to the minimum number of experts required to yield reliable and acceptable cut scores. It has already been suggested that for the Angoff method, a panel of at least 15 experts is required to obtain reliable and trustworthy cut scores [2]. If a smaller panel of experts can be shown to produce reliable cut scores using the EZ method, then the EZ method might be a more convenient, cost-effective, and acceptable solution for reliably setting examinations’ cut scores.

### Objectives

The objective of this study was to estimate the minimal number of panelists required for obtaining acceptable and reliable cut scores for an OSCE using the EZ method.

## Methods

### Ethics statement

The conduct of the study was approved by the Institutional Review Board of Taipei Veterans General Hospital (ref: 2018-01-006CC). Informed consent was exempted for this minimal-risk research.

### Study design

This is a statistical simulation study based on primary data collection (Dataset 1).

### Setting

The primary data for this study were retrieved from a standard-setting exercise, which set cut scores for mock OSCEs conducted at Taipei Veterans General Hospital in 2019 for final-year medical students. The procedure of the primary data generation was described in a previous publication [8], and the method of calculating the cut score is described in the introduction above. The primary data was generated from 31 panelists who assessed the 12 stations of an OSCE, and each expert reported “without any doubt” the highest failure mark and the lowest pass mark for each station.

### Participants

The current study used only simulated data. However, the participants who contributed the primary data were 31 senior clinicians (specialists) working at Taipei Veterans General Hospital. These clinicians had diverse professional backgrounds, including but not limited to pathology, general practice, medicine, obstetrics & gynecology, pediatrics, surgery, rehabilitation, and psychiatry. All participants commonly engaged with medical student clinical assessments. It is noteworthy that the participants only assessed the difficulty of the stations; they did not examine the students in the OSCE stations.

### Variables

The primary data included the highest failure marks and the lowest pass marks for each of the 12 OSCE stations, as advised by each of the panelists.

### Data sources/measurement

For the current study, 1,000 randomly sampled (random, uniform re-sampling method with replacement) samples of panels were generated from the primary data, each comprising 5–25 panelists. Then, for each sampled panel, the cut scores, z-scores, X_H_, X_L_, SE_H_, and SE_L_ were calculated from the EZ method responses, following the procedure described in the Introduction. The statistical analysis used the simulated data only—that is, the X_H_, X_L_, SE_H_, and SE_L_ generated from the simulated panels and the panel sample sizes.

### Bias

No known bias in the primary or the simulated data was identified, nor is there any theoretical reason to assume any bias in such a study.

### Study size

The simulation consisted of 1,000 sub-samples extracted from the primary dataset. This simulated sample size is acceptable in simulation studies [11].

### Statistical methods

The first analysis explored the association between the mean z-score yielded by each panel and the panel size (using visual presentation). This analysis was conducted for each station separately. Next, analysis of variance (ANOVA) was employed to measure whether the differences in the cut scores set by the sampled groups, across all sizes within each station, presented statistically significant differences.

## Results

**Fig. 2.**
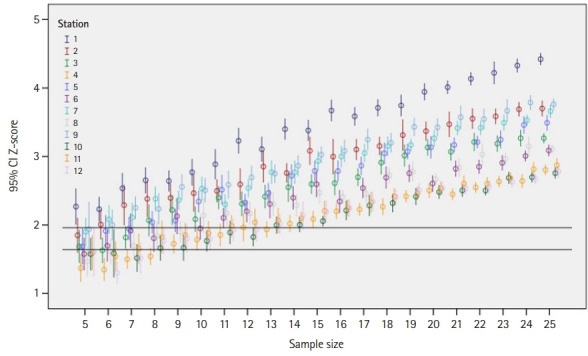
Mean and 95% confidence interval (CI) of Z-scores by panel size for each station.

**Fig. 3.**
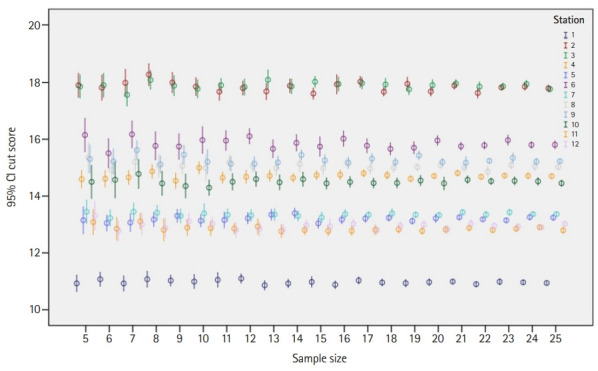
Mean cut score and 95% confidence interval (CI) by panel size for each station.

The number of re-samples per station per sample size ranged from 19 to 51. The results of this study demonstrated that a panel of 10 experts or more yielded cut scores with 1-sided confidence equal to or more than 90% (z-score ≥1.64), and a panel of 15 experts yielded cut scores with 1-sided confidence equal to or more than 95% (z-score ≥1.96) (Fig. 2). The impact of panel size on the cut score was found to be insignificant when the panel size was 5 to 25 (Table 1 and Figs. 2, 3). Specifically, ANOVA showed no significant differences in the cut scores between different panel sizes across all stations (P≥0.243) (Table 1).

## Discussion

### Key results

This study aimed to estimate the minimal number of panelists required for obtaining reliable and trustworthy cut scores when using the EZ method for an OSCE [9]. The results suggest that once the panel comprises 10 or more panelists, the panel size has no statistically significant impact on the cut scores yielded (Table 1). This finding is in line with previous studies using simulated and observed data [2]. This finding is not surprising since there is no theoretical or empirical evidence suggesting that the number of panelists is a source of systematic bias in the cut score obtained.

### Interpretation

The main question is, therefore, what is the minimal number of panelists required to generate a reliable cut score when the EZ method is used? The common belief in the literature is that a high level of agreement among the panelists indicates high reliability of the standard-setting exercise [12]. This view has been challenged in a simulation study of the modified Angoff method [2], which demonstrated that only 5.1% of the variance in the cut score precision (deviation of the obtained cut score from the true cuts-core) was attributed to agreement among the panelists. Moreover, it was also demonstrated that the more diverse the panel (in terms of expertise) the more precise the cut scores were.

Unlike other standard-setting methods, the EZ method has a built-in reliability measure: that is, the z-score used for setting the cut score. The z-score is calculated from (1) the SE_L_ and SE_H_ (Equation 2), which are derived from the variances of the means of lowest pass mark (X_H_) and highest fail mark (X_L_), indicating the level of agreement among the panelists; and (2) from the difference between the means of X_H_ and X_L_ (Equation 2), which indicates the range of perceived borderline score range. Consequently, the larger the borderline range (X_H_–X_L_) and the smaller the SE_L_ and SE_H_, the larger the z-score and the higher the confidence that the cut score is neither a pass nor fail mark. Having the confidence that the cut score is neither pass nor fail is a critical measure for any standard-setting exercise since this is the primary objective of setting cut scores (i.e., to identify a score that reliably separates competent from incompetent students) [1].

In the current study, panels with 10 or more panelists reached a confidence of 90% that the cut score was neither pass nor fail. The confidence increased to 95% when 15 or more panelists were employed. These results demonstrate that the EZ method delivers 3 important outcomes: reliable cut scores; a means to assess the reliability (panelist agreement) of these cut scores; and, the level of confidence that these cut scores are neither a pass nor a fail marks. This is an important finding, suggesting that only 10 panelists are required for the EZ method to yield reliable and trustworthy cut scores. In comparison to other standard-setting methods, using 10 panelists to generate a reliable cut score for a 12-station OSCE, all within 1 hour, is a feasible and quick solution, particularly compared to the alternatives.

### Comparison with previous studies

It is acknowledged that the EZ method utilizes a holistic approach since the assessment made by the panelists is at the whole-station (or examination) level, rather than assessing individual items (or assessment criteria), which may be regarded as a limitation. Nonetheless, this practice has been successfully implemented within the Hofstee method [13]. Studies comparing cut scores yielded from the Hofstee and Angoff methods were found to deliver similar cut scores [14]. Similarly, a comparison of the EZ method with the borderline regression method found that the cut scores yielded from both methods were highly correlated (intraclass correlation coefficient ≥0.744), indicating that the EZ method is strongly associated with the actual difficulty of the OSCE stations [8].

### Limitations

An important limitation of this study is related to the re-sampling method, which generated between 19 and 51 panels for each station. These are not high numbers of samples per station. However, since the smaller the re-sampled size, the larger the variance, this limitation means that some of the results presented in Table 1 could potentially yield a higher significance level, had the number of panels per station been increased. This would further strengthen the results, making the current results rather conservative.

### Generalizability and suggestions

Despite the strong evidence presented in this study, further research is needed to assess the generalizability and utility of the EZ method across different contexts, types of examinations, and populations.

### Conclusion

This study demonstrates that the EZ method is valid and sufficiently reliable for yielding trustworthy cut scores when at least 10 panelists are employed. The EZ method is feasible as it requires less than an hour for a panel to assess 12 OSCE stations (equal to an examination of about 120–180 items), and calculating the cut score requires only basic technical skills. Therefore, the EZ method is proposed as an easy method for setting reliable cut scores for high-stakes examinations, particularly when the availability of panel experts is limited.

## Figures and Tables

**Table 1.** Cut scores by station and panel size

Panel size	Station no.											
	1	2	3	4	5	6	7	8	9	10	11	12
5	10.93	17.90	17.86	14.60	13.15	16.15	13.45	15.35	15.30	14.50	13.08	13.31
6	11.07	17.82	17.90	14.61	13.05	15.51	13.23	14.91	15.22	14.57	12.85	12.78
7	10.93	17.99	17.56	14.65	13.07	16.17	13.45	15.20	15.62	14.78	13.11	13.01
8	11.07	18.28	18.09	14.87	13.17	15.77	13.41	15.07	15.12	14.45	12.81	12.88
9	11.03	18.00	17.88	14.54	13.30	15.74	13.30	15.06	15.46	14.35	12.89	13.11
10	10.99	17.85	17.78	15.00	13.13	15.97	13.39	14.99	15.20	14.30	12.87	13.02
11	11.05	17.67	17.90	14.65	13.16	15.95	13.33	15.18	15.14	14.50	12.86	12.81
12	11.10	17.80	17.84	14.68	13.22	16.11	13.32	15.06	15.14	14.60	12.93	12.85
13	10.86	17.68	18.09	14.71	13.34	15.66	13.35	14.99	15.19	14.48	12.76	12.82
14	10.93	17.88	17.86	14.64	13.40	15.87	13.30	15.13	15.44	14.59	12.80	12.97
15	10.98	17.61	18.02	14.74	13.04	15.74	13.25	14.99	15.26	14.45	12.78	12.93
16	10.88	17.93	17.95	14.75	13.17	16.02	13.37	15.18	15.17	14.50	12.78	13.02
17	11.03	18.02	17.97	14.80	13.21	15.77	13.34	14.97	15.32	14.46	12.81	12.80
18	10.96	17.67	17.93	14.73	13.23	15.66	13.39	15.00	15.19	14.46	12.83	12.95
19	10.94	17.95	17.76	14.61	13.12	15.70	13.34	15.02	15.43	14.55	12.77	13.00
20	10.97	17.68	17.90	14.71	13.21	15.96	13.33	15.09	15.20	14.45	12.82	12.82
21	10.99	17.89	17.96	14.81	13.25	15.75	13.43	15.00	15.18	14.57	12.88	13.02
22	10.90	17.63	17.85	14.68	13.18	15.79	13.35	14.87	15.24	14.52	12.80	12.95
23	10.99	17.82	17.86	14.72	13.15	15.97	13.43	15.09	15.34	14.54	12.85	12.93
24	10.96	17.85	17.94	14.71	13.26	15.80	13.36	15.05	15.21	14.52	12.90	12.90
25	10.94	17.79	17.77	14.70	13.24	15.81	13.37	15.02	15.23	14.45	12.79	13.02
P-value^a)^	0.967	0.243	0.752	0.270	0.702	0.288	0.988	0.932	0.343	0.985	0.768	0.358

a) P-value for cut scores between groups (panel groups) difference taken from the analysis of variance analysis.

## Footnotes

**Data availability**
Data files are available from Harvard Dataverse: https://doi.org/10.7910/DVN/SJNBSW Dataset 1. The primary data generated from the simulated data set.

## Supplementary materials

Supplement 1. Audio recording of the abstract.
